# Food intake during the previous 24 h as a percentage of usual intake: a marker of hypoxia in infants with bronchiolitis: an observational, prospective, multicenter study

**DOI:** 10.1186/1471-2431-13-6

**Published:** 2013-01-11

**Authors:** François Corrard, France de La Rocque, Elvira Martin, Claudie Wollner, Annie Elbez, Marc Koskas, Alain Wollner, Michel Boucherat, Robert Cohen

**Affiliations:** 1 ACTIV (Association Clinique et Thérapeutique Infantile du Val de marne), 27 rue d’Inkermann, 94100, Saint Maur des fossés, France; 2 Physiology Lung Function Department Armand-Trousseau Hospital, Paris, France; 3 Department of Microbiology, CHI Créteil, 40 avenue de Verdun, Créteil, France

**Keywords:** Bronchiolitis, Hypoxia, Feeding, Infant, Out-patient, Intercostal retraction, Subcostal retraction, Supracostal retractions, Respiratory syncytial virus

## Abstract

**Background:**

Hypoxia associated with bronchiolitis is not always easy to assess on clinical grounds alone. The aim of this study was to determine the value of food intake during the previous 24 hours (bottle and spoon feeding), as a percentage of usual intake (24h FI), as a marker of hypoxia, and to compare its diagnostic value with that of usual clinical signs.

**Methods:**

In this observational, prospective, multicenter study, 18 community pediatricians, enrolled 171 infants, aged from 0 to 6 months, with bronchiolitis (rhinorrhea + dyspnea + cough + expiratory sounds). Infants with risk factors (history of prematurity, chronic heart or lung disorders), breast-fed infants, and infants having previously been treated for bronchial disorders were excluded.

The 24h FI, subcostal, intercostal, supracostal retractions, nasal flaring, respiratory rate, pauses, cyanosis, rectal temperature and respiratory syncytial virus test results were noted. The highest stable value of transcutaneous oxygen saturation (SpO2) was recorded. Hypoxia was noted if SpO2 was below 95% and verified.

**Results:**

24h FI ≥ 50% was associated with a 96% likelihood of SpO2 ≥ 95% [95% CI, 91–99]. In univariate analysis, 24h FI < 50% had the highest odds ratio (13.8) for SpO2 < 95%, compared to other 24h FI values and other clinical signs, as well as providing one of the best compromises between specificity (90%) and sensitivity (60%) for identifying infants with hypoxia. In multivariate analysis with adjustment for age, SpO2 < 95% was related to the presence of intercostal retractions (OR = 9.1 [95% CI, 2.4-33.8%]) and 24h FI < 50% (OR = 10.9 [95% CI, 3.0-39.1%]). Hospitalization (17 infants) was strongly related to younger age, 24h FI and intercostal retractions.

**Conclusion:**

In practice, the measure of 24 h FI may be useful in identifying hypoxia and deserves further study.

## Background

Bronchiolitis is the most common viral infection of the lower respiratory tract in infants under 1 year of age [1]. It affects more than 400 000 infants every year in France [2] and is responsible for tens of thousands of hospital admissions. Respiratory syncytial virus (RSV) is usually responsible for the first episode, but other viruses may also be involved. Bronchiolitis is usually mild, resolving spontaneously within a few days, but some infants, especially the very young, develop severe forms with hypoxia and may need hospitalization, primarily for supplemental oxygen administration. Treatment is solely symptomatic, and recent studies have shown that physiotherapy is not beneficial [3,4]. Diagnosis of this infection is often easy, but its severity (hypoxia due to broncho-bronchiolar obstruction) may be difficult to assess. Suckling is a muscular effort that must be coordinated with breathing, and this is more difficult when breathing is restricted by airway obstruction, therefore, reduced feeding might represent a marker of severity.

The amount of food ingested in the past 24 hours is relatively easy to measure accurately, whether taken exclusively from a (graduated) bottle or partly by spoon.

Hypoxia can be diagnosed by non-invasive transcutaneous measurement of oxygen saturation (SpO2). Although many inexpensive devices are available, few are suitable for young infants, who require specific, often costly probes that can be difficult to disinfect. In addition, isolated SpO2 values may not be predictive of subsequent outcome.

The aims of this study were to determine whether food intake in the previous 24 hours, expressed as a percentage of usual intake (24h FI), might serve as an initial screening tool for hypoxia in infants with bronchiolitis, and to analyze the relation between both 24h FI and standard clinical signs and the decision to hospitalize the infant. This tool might be useful for telephone triage or in settings where the history can be used to guide further evaluation.

## Patients and methods

This observational, prospective, multicenter study included infants aged from 0–6 months diagnosed with bronchiolitis (rhinorrhea + cough + dyspnea + expiratory breath sounds) during the three winter months (November to January) when the infection is most frequent in Europe. Infants were excluded if they had risk factors (history of prematurity, chronic heart or lung disease), were breast-fed (even partially), or had previously received treatment for a bronchial disorder (bronchodilators, corticosteroids, physiotherapy). Infants cared for outside the home by child minders could be enrolled, provided the parents knew the precise amount of food ingested in the previous 24 hours. Food intake was calculated as the sum of bottle and spoon feeding. The infants were recruited by 18 community pediatricians in the Paris region.

### Data collection

The pediatrician noted the infant’s age, the duration (days) of wheezing, any fever or loose stools in the previous 24 hours, the usual type of feeding, the number of meals taken with a bottle or spoon, and the amount of formula-milk usually drunk, per meal, then calculated the total volume of milk taken over the past 24 hours. If the baby was partly spoon-fed, the pediatrician noted any change in the amount ingested during the previous 24 hours compared to normal.

Quiet, non crying infants were examined for retractions (subcostal, intercostal, suprasternal, and nasal flaring), the respiratory rate (recorded for one minute; rapid if >50/min), cyanosis, and respiratory pauses (during the examination or reported by the parents). SpO2 was measured only after collecting this information, in order not to interfere with the clinical examination. Rectal temperature was routinely measured.

The pediatricians used a Hellcor pulse oximeter to measure SpO2. The prolonged measurements took into account the pulsatile nature of arterial flow (displayed on the device), as well as the highest SpO2 value recorded (sources of error tend to give lower results) and its stability. Hypoxia was defined as an SpO2 below 95%. All results below < 95% were verified by a second measurement, and only the highest value was recorded.

RSV was sought routinely (immunochromatographic screening test VRSTOP+® (ALL.DIAG SA, Strasbourg, France) with nasal swabs).

The doctor recorded the decision regarding immediate hospitalization. The hospital reports were recovered later to determine whether the child had received specific hospital care (oxygen, infusion, or gastric gavage).

### Statistical analysis

The sensitivity, specificity, positive predictive values (PPV), negative predictive values (NPV), positive likelihood ratios (LR^+^) and negative likelihood ratios (LR^-^) were calculated with their 95% confidence intervals, taking an Sp02 of < 95% as reference, and ROC curves were computed. Means were compared between groups by using a t-test with an unequal variance option if necessary, and percentages were compared by using the chi2 test or Fisher's exact test, as appropriate. Significance was assumed at p < 0.05. Univariate and multivariate analyses (logistic regression) were used to identify factors associated with SpO2 < 95% after adjustment for age (< or ≥ 2 months), and odds ratios (OR) were calculated with their 95%CI. Stata SE 9.1 statistical software was used.

### Ethics

A poster placed in the waiting room invited parents to participate in the study and informed them that they were free to refuse their participation. They were informed that the study was anonymous and would have no influence on the care received by their child. Furthermore, before enrolment, the investigators informed the parents about the purpose of the study and requested their oral consent. During the period in which this observational study was performed, French legislation did not require ethical approval or other authorizations for research not involving unusual or additional procedures relative to usual practice.

## Results

During three winter periods, from late 2006 to early 2009, the participating pediatricians recruited 171 infants with a mean age of 1.6 ± 3.7 months (median 4 months). The infants were seen between the first and sixth day after the onset of chest sounds (average 2 days, ±1.5 days), as reported by the parents.

During the previous 24 hours, 22% of the infants had been febrile (≥ 38°C) (21% were febrile at the time of the examination), and 14% had had three or more loose stools.

Feeding consisted exclusively of formula-milk in 87% of infants, while the remaining 13% of infants, all aged more than 4 months, were partly spoon-fed. Food intake in the previous 24 hours (24h FI), as a percentage of the usual amount, was less than 50% in 14% of cases, ≥ 50% to < 70% in 26% of cases, and ≥ 70% in 60% of cases.

Nasal flaring and suprasternal, intercostal or subcostal retractions were present in respectively 2%, 15%, 25% and 30% of infants.

The respiratory frequency was ≥ 50/min in 43% of infants and ≥ 60/min in 23% of infants.

Cyanosis was noted in 5 infants, one of whom also had breathing pauses.

Mean oxygen saturation was 98% ± 3% (80% to 100%; median 98%). Fifteen infants (9%) had SpO2 < 95% and 2 infants had SpO2 < 90%.

RSV was tested for in 104 infants, of whom 42% were positive.

Seventeen of the 171 infants were hospitalized immediately after the examination. Ten of the hospitalized infants received specific hospital care, consisting of oxygen in 9 cases, gastric gavage in 6 cases and infusion in 5 cases.

### Relation between SpO2 and 24h FI

**Figure 1:**
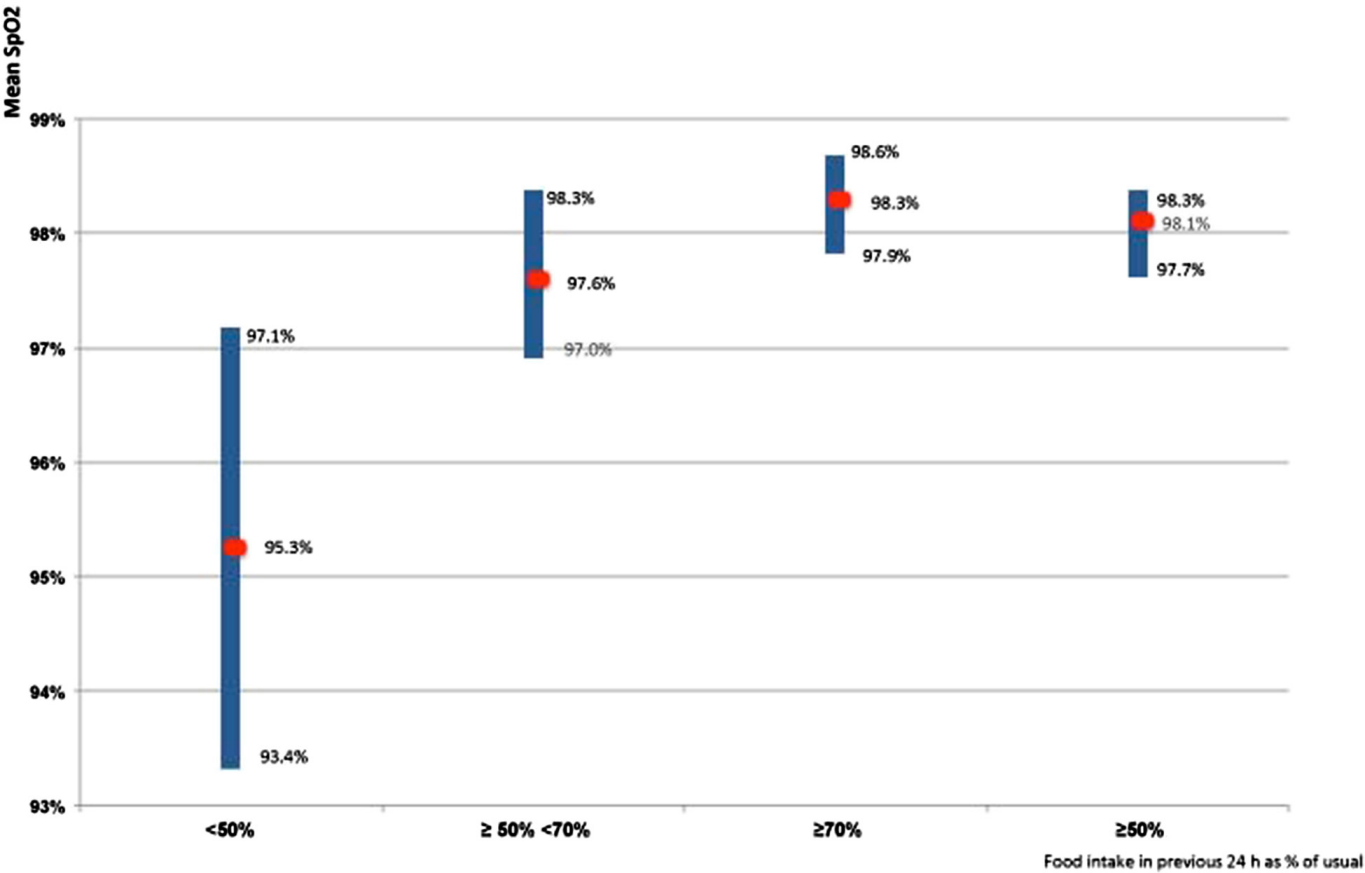
**Mean SpO**_**2**_**values according to food intake in the previous 24 h (as a percentage of usual intake).** The three left-hand rectangles show that mean SpO2 values vary in the same direction as 24 h % food intake. The first and last rectangles show the significant difference in mean SpO2 values between infants who took < 50% or ≥ 50% of their usual volume of food in the previous 24 h (4 missing data). Length of the rectangles according to the 95% confidence intervals.

The closer food intake in the previous 24 hours was to the usual amount, the closer SpO2 was to normal: higher 24h FI values were associated with higher SpO2 (Figure 1). The average SpO2 ininfants whose food intake fell by more than 50% was 95.5% [95% CI, 93.6-97.4%], compared to 98.1% [95% CI, 97.7-98.3%] in the infants whose food intake remained higher than 50% of normal (p < 0.001). In the latter group, SpO2 was normal (≥ 95%) in 96% of cases [95% CI, 91-99%].

When food intake during the previous 24 hours was 50% or more of the usual amount, the likelihood that saturation would be normal (SpO2 ≥ 95%) was 96% [95% CI, 91%-99%].

### Relation between SpO2, clinical status and food intake

**Figure 2:**
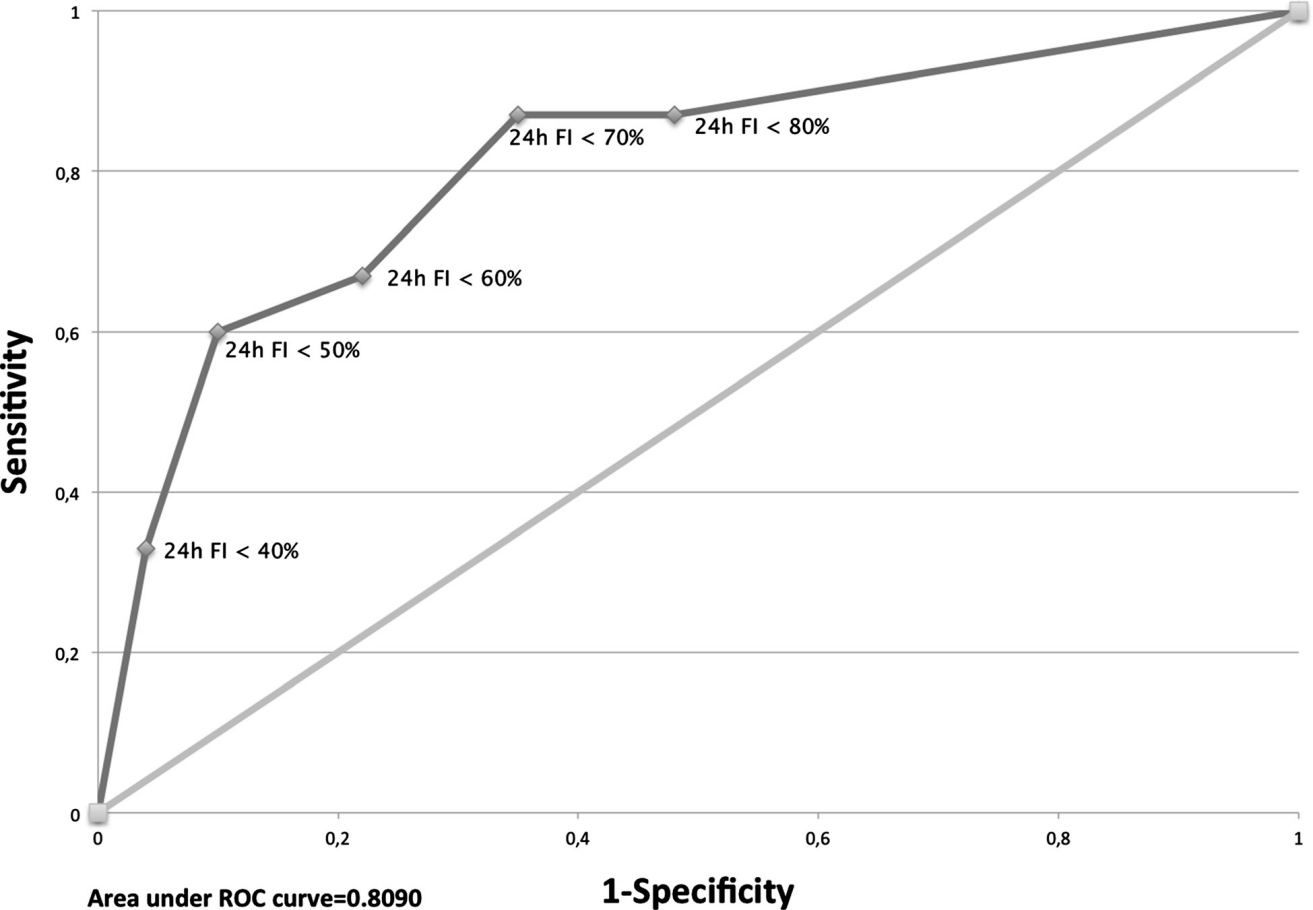
Roc curve : Compromise sensibility / specificity for different values of 24h FI.

The positive predictive values (PPV) of 24h FI for SpO2 < 95% were highest with cutoffs of < 40% and < 50% (Table 1). However, 24h FI < 50% had the highest specificity (90%) and moderate sensitivity (60%) (Figure 2), and also the highest odds ratio of all 24h FI cutoffs in univariate analysis (Table 1).

**Table 1:** **Sensitivity, specificity, PPV, NPV, LR**^**+**^**, LR**^**-**^**and odds ratios of clinical signs of hypoxia (SpO2 < 95%) in infants with bronchiolitis**

	**Sensitivity % [95% IC]**	**Specificity % [95% IC]**	**PPV % [95% IC]**	**NPV % [95% IC]**	**LR**^**+**^**n [95% IC]**	**LR**^**-**^**n [95% IC]**	**Odds ratio n [95% IC]**
Suprasternal retraction	50 [21–79]	87 [81–92]	24 [9–45]	96 [91–98]	4 [2-8]	0,6 [0.3 - 1]	6.9 [2.0 - 23.7]
Intercostal retraction	73 [45–92]	80 [73–86]	26 [14–42]	97 [92–99]	3.6 [2-6]	0.3 [0.1 - 78]	10.8 [3.2 - 36.3]
Subcostal retraction	47 [21–73]	72 [64–79]	14 [6–27]	93 [87–97]	1.7 [0.9 - 3]	0.7 [0,5 – 1.2]	2.3 [0.8 - 6.6]
Polypnea (≥ 50/min)	87 [60–98]	62 [53–70]	19 [10–30]	98 [93–100]	2.3 [1.7 - 3]	0.2 [0.06 – 0.8]	10.5 [2.3 - 48.2]
24h FI < 70%	87 [60–98]	65 [56–72]	19 [11–31]	98 [93–100]	2.4 [1.8 – 3.2]	0.2 [0.06 – 0.8]	11.8 [2.6 - 54.2]
24h FI < 60%	67 [38–88]	78 [70–84]	23 [11 \- 38]	96 [91–99]	3 **[1.9 – 4.7]**	0.4 [0.2 – 0.8]	6.9 [2.2 - 21.7]
**24h FI < 50%**	**60** [32–84]	**90** [84–94]	**38** [19–59]	**96** [91–99]	**6.1**[3-11]	**0.4** [0.2 – 0.8]	**13.8** [4.3 - 44.1]
24h FI < 40%	33 [12–62]	96 [92–99]	46 [17–77]	94 [89–97]	8.5 [2.9 - 24]	0.7 [0.5 – 0.99]	12.2 [3.2 - 47.2]

*24h FI* food intake in previous 24h as a % of usual intake; *PPV* positive predictive value ; *NPV* negative predictive value ; *LR*^*+*^ positive likelihood ratio ; *LR*^*-*^ negative likelihood ratio; *[95% CI]* 95% confidence interval.

In multivariate analysis, the only variables significantly associated with SpO2 < 95% after adjustment for age were intercostal retraction (OR = 9.1 [95% CI 2.4 to 33.8]) and 24h FI < 50% (OR = 10.9 [95% CI 3.0 to 39.1]).

### 24h FI and hospitalization

The 17 hospitalized infants had significantly lower SpO2 values (94% ± 5% versus 98% ± 2%, p < 0.005) and significantly lower 24h FI values (56% ± 24% versus 76 ± 19%, p < 0.0003) than the non-hospitalized infants.

Nine of the 17 hospitalized infants (Table 2) had both 24h FI < 50% and SpO2 < 95%, while 24h FI was ≥ 50% in 90% of the non-hospitalized infants (p < 0.001). Six hospitalized infants had 24h FI > 50% and no obvious signs of respiratory distress, but all were very young (< 2 months). Four of these six infants did not require specific hospital care. The fifth child had a 24h FI value very close to the cutoff (52%), with rapid breathing, intercostal retractions and an SpO2 of 92%. The sixth child had a 24h FI of 67% and an SpO2 of 92%, but also had high fever (39.6°C) during the previous 24 hours and rapid breathing (55 breaths/min), probably due to fever. This infant did not require specific hospital care.

**Table 2:** Factors associated with hospitalization for bronchiolitis

	**Hospitalized (n = 17)**		**Non hospitalized (n = 154)**		
	**n**	**%**	**n**	**%**	**p**
Age < 2 months	13	76%	7	5%	< 0.001
24h FI < 50%	9	53%	15/150 (*)	10%	< 0.001
Intercostal retraction	9	53%	33/151 (*)	22%	< 0.005
1 or more of these 3 factors	14	82%	49/147 (*)	33%	< 0.001
SpO2 < 95%	11	65%	4	3%	< 0.001

* missing data.

In multivariate analysis, the decision to hospitalize was very strongly associated with Sp02 < 95% (p < 0.0001) and age < 2 months (p = 0.001), but not with the other parameters. However, when saturation was excluded, the decision to hospitalize was strongly associated with age < 2 months (OR = 14.7 [95% CI 3.1 to 69.8]), 24h FI < 50% (OR = 10.6 [95% CI 3.0 to 37.3]) and intercostal retractions (OR = 3.4 [95% CI 1.0 to 11.4]). Thus, if SpO2 measurement had not been available, the decision to hospitalize could have been based on age < 2 months, 24h FI < 50%, and intercostal retractions.

## Discussion

This study suggests that recent changes in food intake by infants with bronchiolitis closely reflect their oxygenation status. The 24h FI cutoff most strongly associated with hypoxia (odds ratio = 13.8 [4.3 to 44.1]) was 50% of the usual amount, and this cutoff also had high negative predictive value. In the study population, there were 14 infants who took less than 50% of their usual food intake, and only one of them was not hypoxic. This 50% cutoff also represented the best compromise (moderate sensitivity, high specificity) for identifying infants with SpO2 < 95%. In addition, multivariate analysis showed that this parameter remained correlated with SpO2 < 95%, even after adjustment for age and intercostal retractions, while the other clinical and biological parameters showed no significant correlation with SpO2. This cutoff (50%) would have two advantages. First, if food intake during the past 24 hours remains more than half the usual amount, the likelihood of normal oxygenation status is 96% (or at least 91%, the lower limit of the confidence interval), indicating that bronchiolar obstruction is well tolerated and that pulse oximetry is probably unnecessary in the absence of associated gravity signs. In contrast, food intake below 50% of normal calls for pulse oximetry and medical attention.

This 50% cutoff has a certain tolerance: if 24h FI is 40%, then the negative predictive value only falls from 96% to 94% and the odds ratio from 13.8 to 12.2. The clinical implications therefore remain valid.

24h FI might be used as a screen to further testing. This might be useful for telephone triage or other settings where the history can be used to guide further evaluation, as monitoring a bronchiolitic child in the home.

Food intake has already been included in severity scores and guidelines, but only as “reduced or normal” [2,5-7], or “good, reduced or poor” [8-10]. The November 2006 Scottish guideline [11] mentioned a < 50% reduction in fluid intake in the previous 24 hours as a gravity sign, but this was based on expert opinion and not on published research.

Physiological studies of milk intake by infants with bronchiolitis [12] have shown that the number of sucking and swallowing actions is the same as in healthy infants, but that milk flow during these two movements is reduced. In addition, swallowing movements are followed less by expiration and more by inspiration, and periods of apnea are twice as frequent after swallowing. Suckling is a muscular effort that must be coordinated with breathing, and this is more difficult when breathing is restricted by airway obstruction, when the child is tired, and when the muscles lack oxygen [13].

Parents are generally aware of the amount of food their infant usually consumes, and can accurately recall food intake in the previous 24 hours, based on the amount prepared and the amount left, especially when their child is sick. Indeed, parents prepare the same quantity of milk (powder scoops and bottle graduations) each day for a given meal. The amount left in the bottle by healthy infants does not vary much, but increases during bronchiolitis. Thus, in practice, 24-hour percentage food intake is easy to calculate.

In theory, SpO2 would be an accurate marker of hypoxia, which is directly related through the hemoglobin dissociation curve. Hypoxia is the main complication of bronchiolitis. However, SpO2 is tricky to measure, requiring a considerable period of calm, and a correctly recorded arterial pulse. Correct sensor positioning may take several attempts. In addition, pediatric sensors are fragile, difficult to disinfect correctly, and expensive for single use. In the range of values generally measured in this setting (85-100%), a small change in SpO2 is associated with a large change in PaO2. The result is also observer-dependent and may vary over a 30-minute interval [14]. This is why we verified all values below < 95% by a second measurement, and only used the highest value.

The 95% SpO2 cutoff is commonly used to define hypoxia, and oxygen therapy is recommended if SpO2 is < 90% for a prolonged period in an infant not at risk (term birth, no heart or lung disease and age more than 3 months) [5].

Among the three types of retraction (suprasternal, intercostal and subcostal), intercostal retractions were most closely associated with SpO2 < 95%. Wang [13] reported that the appreciation of retractions is observer-dependent, even when the observer is a healthcare professional, and that the appreciation may vary between two separate observations made 10 to 30 minutes apart. The degree of retractions is difficult to quantify, especially in borderline situations. In addition, they are difficult for parents to assess without specific training. Retractions are included in most scores, but these are not always easy to use in daily practice. Measurement of 24 hours percentage food intake seems to be far simpler for parents.

Hospitalization was associated with hypoxia and with age < 2 months (French guidelines recommend hospitalization of all bronchiolitic infants less than 6 weeks old). Hypoxia was associated with 24h FI < 50% and intercostal retractions. In the physician’s office, if SpO2 cannot be measured, these two signs, especially 24h FI < 50%, in addition to age, can help to decide whether hospitalization is necessary.

This study may have certain limitations. First, the same pediatrician assessed all the data (24h FI, clinical signs of respiratory impairment, oxymetry) and took the decision regarding hospitalization. We tried to limit the subjective element by establishing the order of data collection and applying precautions during oxymetry.

Second, these results are applicable to patients presenting to community practices. Further studies of sicker infants (emergency departments, hospital inpatients) would be necessary to determine whether 24h FI remains valid in this population. It should be noted that most studies of severe adverse outcomes in this setting have involved inpatients, which limits their relevance to other situations [5]. Finally, we excluded breast-feeding infants, as the amount of milk ingested could not easily be estimated, notably from the suckling time [15].

## Conclusion

We studied the semiological value of a clinical sign for assessing the severity of bronchiolitis in previously healthy, mainly bottle-fed, full-term infants presenting to a pediatrician’s office.

Food intake during the previous 24 hours, as a percentage of usual intake, was predictive of oxygenation status (hypoxia or normoxia), and is easy for parents to memorize and measure. Apart from in very young infants (less than 6 weeks old), this parameter could serve as an initial screening tool and to monitor bronchiolitic child in the home.

If an infant has ingested at least half the usual amount of food during the previous 24 hours, hypoxia is unlikely; in contrast, if food intake falls below half the usual amount, medical attention is required.

French version of the manuscript : “see Additional file 1”.

## Abbreviations

24h FI: Food intake during the previous 24 hours as a percentage of usual intake; ACTIV: Association Clinique et Thérapeutique Infantile du Val de marne, a research institute on pediatric community acquired infections; CI: Confidence interval; LR^+^: Positive likelihood ratio; LR^-^: Negative likelihood ratio; NPV: Negative Predictive Value; OR: Odds ratio; PPV: Positive predictive value; RSV: Respiratory Syncitial Virus; SpO2: Saturation of hemoglobin with oxygen as measured by pulse oxymetry.

## Competing interests

All the authors declare that no financial support for the submitted work was received from anyone other than their employer; that they have no financial relationships with commercial entities that might have have an interest in the submitted work in the previous 3 years; that they have no spouses, partners, or children with financial relationships that may be relevant to the submitted work; and that they have no non-financial interests that may be relevant to the submitted work.

This work was submitted by ACTIV. The funders of ACTIV had no role in the study design, data collection or analysis, the decision to publish, or the preparation of the manuscript.

## Authors’ information

François Corrard is a French pediatric and President of a research institute on pediatric community acquired infections (ACTIV). His main research interests are epidemiologic studies and clinical trials in community acquired infections, including pneumococcal diseases, the rhinopharyngeal flora, and vaccines. He has published more than 10 papers in English.

## Authors’ contributions

Designed the study: FC FLR EM CW AE MK AW MB RC. MB designed the data base. Enrolled patients: FC CW AE MK AW RC. Analysed the data: FC FLR EM MK RC. Contributed to the writing of the paper: FC EM MK RC. Agree with the manuscript’s results and conclusions: FC FLR EM AE MK AW MB RC. All authors read and approved the final manuscript.

## Pre-publication history

The pre-publication history for this paper can be accessed here:

http://www.biomedcentral.com/1471-2431/13/6/prepub

## Supplementary Material

Additional file 1 Bronchiolite et prise alimentaire des dernières 24 heures: un outil de dépistage de l’hypoxie (The French version of the manuscript). Click here for file
