# Supplementary material for: Food intake during the previous 24 h as a percentage of usual intake: a marker of hypoxia in infants with bronchiolitis: an observational, prospective, multicenter study
Source: BMC Pediatr. 2013 Jan 11;13:6. doi: 10.1186/1471-2431-13-6 (PMC3557207; doi:10.1186/1471-2431-13-6)
Supplement: Additional file 1 — Bronchiolite et prise alimentaire des dernières 24 heures: un outil de dépistage de l’hypoxie (The French version of the manuscript). [file 1471-2431-13-6-S1.doc]

Bronchiolite et prise alimentaire des dernières 24 heures : un outil de dépistage de l’hypoxie

François Corrard, France de La Rocque, Elvira Martin, Claudie Wollner, Annie Elbez, Marc Koskas, Alain Wollner, et Robert Cohen.

Introduction

La bronchiolite est l’infection virale respiratoire basse la plus fréquente de l’enfant de moins de 1 an (1). Elle touche tous les ans en France plus de 400 000 nourrissons (6) et est responsable de dizaine de milliers d’hospitalisation. Le virus respiratoire syncytial (VRS) est le virus le plus souvent responsable du premier épisode mais d’autres virus sont aussi impliqués. C’est une maladie généralement bénigne qui guérit spontanément en quelques jours, cependant certains nourrissons en particulier les plus jeunes peuvent développer des formes sévères, hypoxémiante justifiant l’hospitalisation essentiellement pour l’administration d’oxygène. En effet, il n’existe aucun traitement spécifique, les traitements proposés sont largement symptomatiques, et des études récentes confirment l’absence d’intérêt de la kinésithérapie (2-3). La difficulté est moins de poser le diagnostic, facile à la phase d’état, que d’évaluer sa gravité, l’hypoxémie, dépendante de l’obstruction broncho-bronchiolaire. Elle pourrait être approchée, dans ce contexte difficile d’efforts musculaires respiratoires permanents, par la capacité à réaliser simultanément un autre effort, celui de se nourrir.

La quantité alimentaire ingérée les dernières 24 heures est une mesure relativement facile et précise quand elle est prise au biberon (graduations) et même lorsque l’enfant commence à utiliser partiellement sa cuillère.

L’hypoxémie peut être évaluée à partir d’une mesure transcutanée (SpO2) non invasive de la saturation en oxygène. Si de nombreux appareils peu coûteux sont disponibles, peu sont adaptés aux jeunes nourrissons qui nécessitent des sondes spécifiques souvent coûteuses et dont les conditions de désinfections ne sont pas toujours aisées à respecter. De plus la mesure de la saturation à un moment donné ne préjuge pas de l’évolution ultérieure de la maladie.

L’objectif de cette étude est de déterminer l’intérêt de la mesure du pourcentage de la prise alimentaire des dernières 24 heures par rapport à la quantité habituellement ingérée (Alim 24h) comme outil de premier dépistage de l’hypoxémie, d’étudier sa valeur par rapport aux autres signes cliniques et d’évaluer sa pertinence pour décider d’hospitaliser un enfant.

Patients et Méthode

Dans cette étude observationnelle, prospective et multicentrique, étaient inclus les enfants de 0 à 6 mois, présentant une bronchiolite (rhinorrhée + toux + dyspnée expiratoire bruyante) pendant les trois mois d’hiver (Novembre à Janvier) où cette maladie sévit le plus en Europe. Etaient exclus les enfants présentant des facteurs de risque (ancien prématuré, maladie chronique cardiaque ou pulmonaire), les enfants nourris au sein, même partiellement, les enfants ayant déjà reçu un traitement à visée bronchique (bronchodilatateur, corticothérapie, kinésithérapie). Les enfants gardés hors du domicile par une assistante maternelle pouvaient être inclus à condition que les parents aient une connaissance précise de la quantité alimentaire ingérée dans les dernières 24 heures. La prise alimentaire comprenait celle des biberons, complétée pour les plus grands enfants par celle des repas pris à la petite cuillère.

Les enfants étaient recrutés par 18 pédiatres libéraux, dans la région parisienne.

Le recueil des données

Le médecin relevait l’âge de l’enfant, la durée en jours de la respiration bruyante, l’existence dans les 24 dernières heures de fièvre et de selles liquides, détaillait le mode habituel d’alimentation, le nombre de repas pris au biberon et à la petite cuillère, notait la quantité de lait bue habituellement et additionnait la quantité des repas pris au cours des dernières 24 heures. Si l’enfant s’alimentait à la petite cuillère, il était noté la variation de quantité prise dans les dernières 24 heures par rapport à d’habitude.

L’examen chez un enfant calme, ne pleurant pas, relevait la présence d’un tirage (sous-costal, intercostal, sus-sternal, battements des ailes du nez), la fréquence respiratoire comptée sur une minute (polypnée si > 50/mn), recherchait l’existence d’une cyanose, de pauses respiratoires (à l’examen ou rapportées par les parents). Ce n’est qu’après avoir recueilli ces renseignements, que la saturation (SpO2) était mesurée afin de ne pas influencer l’observation clinique. La température rectale était prise systématiquement.

Chaque pédiatre disposait d’un saturomètre de marque Hellcor. La mesure prolongée tenait compte de la qualité du caractère pulsatile du sang artériel (noté sur l’appareil), de la valeur la plus haute relevée (les causes d’erreur ayant plutôt tendance à diminuer le résultat) et de sa stabilité. Le seuil d’hypoxémie est fixé à 95%. Tout résultat < 95% était vérifié par une deuxième mesure en retenant le résultat le plus élevé.

La présence de VRS était recherchée (test immunochromatographique VRSTOP+ de All.Diag sur écouvillonnage et brossage nasal).

Le médecin mentionnait s’il décidait une hospitalisation immédiate. Le compte rendu était récupéré ultérieurement pour savoir si l’enfant avait bénéficié de soins hospitaliers spécifiques (oxygénothérapie, perfusion ou gavage gastrique).

Statistiques

La sensibilité, la spécificité, la valeur prédictive positive (VPP), la valeur prédictive négative (VPN) et les rapports de vraisemblance positifs et négatifs (RV+ et RV-) ont été calculés avec leurs intervalles de confiance à 95% [IC 95%] en prenant comme référence une saturation Sp02 < 95%. Une courbe ROC a été construite. Les moyennes entre 2 groupes ont été comparées par le t-test avec l’option variance inégale si nécessaire, la comparaison de pourcentages par le test du chi2 ou un test exact de Fisher si nécessaire. Le seuil de significativité correspond à p < 0,05. Des analyses uni et multivariées (régression logistique) ont été réalisées pour évaluer les facteurs liés à une SpO2 < 95% après ajustement sur l’âge (< 2 ou ≥ 2 mois), les odds-ratios (OR) ont été calculés avec leurs IC 95%. Les analyses statistiques sont effectuées avec le logiciel Stata SE 9.1.

Ethique

Une affiche dans la salle d’attente, invitait les parents à participer à l’étude en précisant qu’ils pouvaient refuser. Ils étaient avertis que l’étude était anonyme et qu’elle ne changeait en rien les soins donnés à leur enfant. De plus, avant inclusion, les investigateurs informaient les parents du but de l’étude et recueillaient verbalement leurs consentements. A l’époque ou cette étude observationnelle a eu lieu, la législation française n’imposait pas le recours à un comité d’éthique pour de tels travaux, sans ajout de procédure inhabituelle par rapport à la pratique courante.

Résultats

Lors de trois hivers de fin 2006 à début 2009, 171 enfants ont été inclus, d’âge moyen 3,7 mois ± 1,6 avec une médiane à 4 mois. Ils étaient vus entre le 1^er^ et 6^ème^ jour après l’apparition des bruits pulmonaires audibles par les parents, en moyenne après 2 jours (+/- 1,5 jours).

Dans les dernières 24 heures, 22% étaient fébriles (≥ 38°C) (21% lors de l’examen), 14% avaient eu 3 selles liquides ou plus.

Pour 87% des enfants, le régime était exclusivement lacté, 13%, âgés de plus de 4 mois, prenaient un complément à la petite cuillère. Le pourcentage de quantité alimentaire ingérée dans les dernières 24 heures par rapport à la quantité habituelle (Alim 24h) était strictement inférieur à 50% pour 14% des enfants, ≥ 50% et < 70% pour 26% et ≥ 70% pour 60%.

Un battement des ailes du nez, un tirage sus-sternal, intercostal ou sous-costal étaient présents chez 2%, 15%, 25%, 30% des enfants, respectivement.

La fréquence respiratoire était ≥ 50/mn pour 43% des enfants et ≥ 60/mn pour 23%.

Une cyanose était notée chez 5 enfants et l’un d’entre eux présentait aussi des pauses respiratoires.

La saturation moyenne en oxygène était en moyenne de 98% ± 3 % (de 80 à 100 %), avec une médiane à 98%. Quinze enfants (9%) avaient une SpO2 < 95%, pour 2 enfants la SpO2 était < 90%.

Le test de dépistage du VRS réalisé pour 104 enfants était positif dans 42% des cas.

Sur les 171 enfants, 17 étaient hospitalisés immédiatement après la consultation. Parmi ces enfants hospitalisés, 10 ont reçu des soins spécifiques hospitaliers : 9 de l’oxygène, 6 un gavage gastrique et 5 une perfusion.

**SpO2 et pourcentage de prise alimentaire des dernières 24 heures**

Plus la quantité alimentaire prise au cours des dernières 24 heures se rapprochait de ce que prenait l’enfant habituellement, plus la SpO2 était proche de la normale (Fig. 1).

La SpO2 moyenne des enfants ayant pris < 50% de leur ration habituelle pendant les dernières 24 heures (95,5% [IC 95%, 93,6%-97,4%]) était significativement différente de celle des enfants ayant pris plus 50 % de leur ration alimentaire habituelle (98,1% [IC 95%, 97,7%-98,3%]), p< 0,001.

Si l’enfant a ingéré dans les dernières 24 heures 50% ou plus de sa ration habituelle, la probabilité que sa saturation soit normale (≥ 95%) est de 96% [IC 95%, 91%-99%].

**SpO2 < 95% et signes cliniques et alimentaires**

Les valeurs prédictives positives (VPP) étaient les plus importantes pour Alim 24 h < 40% et pour Alim 24h < 50% (tableau 1). Cependant, Alim 24h < 50% était le paramètre qui présentait une spécificité élevée (90%) avec une sensibilité moyenne (60%) (Fig. 2), ainsi que l’Odd Ratio le plus élevé en univarié (Tableau 1).

L’analyse multivariée montrait que les seules variables significativement liées à une SpO2 < 95%, après ajustement sur l’âge, étaient le tirage intercostal, OR= 9,1 [IC 95% 2,4-33,8] et Alim 24h < 50%, OR= 10,9 [IC 95% 3,0-39,1], les autres variables cliniques et alimentaires n’étaient pas statistiquement significatives.

**Alim 24h et hospitalisation**

Les 17 enfants hospitalisés avaient une saturation significativement plus basse (94% ± 5% *versus* 98% ± 2%, p < 0,005) et une Alim 24h significativement plus basse (56% ± 24% *versus* 76 ± 19%, p < 0,0003) que les non-hospitalisés.

Parmi ces enfants hospitalisés (Tableau 2), plus de la moitié, 9 enfants, avaient à la fois une Alim 24h < 50% et une SpO2 < 95% alors que parmi les enfants non hospitalisés, 90% avaient une Alim 24h ≥ 50% (p < 0,001). Six enfants hospitalisés avaient une Alim 24h > 50% sans détresse respiratoire évidente mais un jeune âge (< 2 mois). Parmi ces 6 enfants, 4 enfants n’avaient pas bénéficié de soins hospitaliers spécifiques. Le 5^ème^ enfant avait une Alim 24h très proche du seuil (52%) mais avec une polypnée, un tirage intercostal et une SpO2 à 92%. Le 6^ème^ enfant avait une Alim 24h à 67% avec SpO2 à 92% mais avec une forte fièvre (39,6°C) depuis 24 heures et une polypnée à 55 (imputable à la fièvre). Son état n’a pas nécessité de soins hospitaliers spécifiques.

La décision d’hospitaliser était très fortement liée à la saturation < 95% (p < 0,0001) et à l’âge < 2 mois (p=0,001)). L’analyse multivariée montrait que les autres paramètres n’étaient pas statistiquement significatifs.

Cependant, si on ne tient pas compte de la saturation, l’analyse multivariée montrait que la décision d’hospitaliser était fortement liée à l’âge < 2 mois (OR= 14,7 [IC 95% 3,1-69,8]), à Alim 24h < 50% (OR= 10,6 [IC 95% 3,0-37,3]) et au tirage intercostal (OR= 3,4 [IC 95% 1,0-11,4]), les autres paramètres n’étant pas statistiquement significatifs. Ainsi, la décision d’hospitaliser était très liée à l’âge de l’enfant < 2 mois et à la saturation < 95%, mais si la mesure de la saturation n’avait pas été possible, la décision d’hospitaliser aurait pu être prise, en plus de l’âge, à la présence d’un tirage intercostal et surtout d’Alim 24h < 50%.

Discussion

Cette étude suggère que l’évolution de la quantité alimentaire ingérée par un nourrisson atteint de bronchiolite est un bon reflet de la qualité de ses échanges respiratoires.

Le seuil de 50% du pourcentage de prise alimentaire, outre sa VPN importante, comporte par rapport aux autres valeurs d’Alim 24h, l’association la plus forte avec l’hypoxémie (Odds Ratio = 13,8 [4,3 - 44,1]. Ainsi, dans cette population, il existe 14 enfants qui prennent moins de 50% que d’habitude et qui sont hypoxémiques pour un enfant normoxémique, dans les mêmes conditions alimentaires. Cette valeur de 50% représente aussi le meilleur compromis (sensibilité moyenne, spécificité élevée) pour dépister les enfants avec une SpO2 < 95%. Par ailleurs, l’analyse multivariée a montré que ce paramètre restait statistiquement significatif même après ajustement sur l’âge et le tirage intercostal, les autres paramètres cliniques ou biologiques n’apparaissant pas significatifs. Cette valeur de 50% comporterait deux intérêts. Si la prise est ≥ 50%, les enfants seraient normoxémiques à 96% (au moins à 91%, limite basse de l’intervalle de confiance) : l’obstruction bronchiolaire est bien tolérée et la mesure de la saturométrie probablement inutile en l’absence de signes de gravité associés. Si la prise est < 50%, c’est la situation la plus pertinente pour dépister l’hypoxémie et déclencher une consultation pour évaluer l’état respiratoire.

Cette valeur de 50% admet en pratique une certaine tolérance d’appréciation. Si Alim 24h n’est que de 40%, la VPN diminue seulement de 96% à 94% et l’Odds Ratio de 13,8 à 12,2. Les implications cliniques restent encore valables.

Alim 24h pourrait permettre un premier tri avant d’autres évaluations, en particulier au téléphone en premier recours ou pour le suivi à distance d’un enfant malade pour lequel le diagnostic de bronchiolite a été posé.

La difficulté alimentaire chez le nourrisson a été déjà prise en compte dans des scores de gravité et des recommandations, mais la quantification était vague, prise diminuée ou non (4-7), bonne, diminuée ou mauvaise (8-10). Les recommandations écossaises de Novembre 2006 (11) mentionnaient comme élément de gravité cette diminution de prise de boisson < 50% dans les dernières 24 heures, mais en s’appuyant sur des avis d’experts sans l’appui d’étude publiée.

L’analyse physiologique de la prise de lait de nourrissons atteints de bronchiolite (12) montrait que le nombre de succion et de déglutition restait le même que pour des enfants bien portants mais que le volume de lait qui transitait lors de ces deux mouvements était diminué. Les déglutitions étaient moins suivies d’expiration et davantage d’inspiration. Les périodes d’apnée étaient deux fois plus fréquentes après déglutition. Téter est un travail musculaire qui doit s’articuler avec la respiration, exercice d’autant plus difficile que la respiration est laborieuse par l’obstruction des voies aériennes, que l’enfant est fatigué et que les muscles sont en hypoxie (13).

En pratique, la mesure de la quantité alimentaire ingérée sur 24 heures est habituellement bien mémorisée par la ou les personnes qui ont nourri l’enfant, en particulier quand celui-ci est malade, que la nourriture soit prise au biberon (graduations) ou que l’enfant commence à utiliser partiellement sa cuillère. Elle est calculée par différence entre la quantité préparée et la quantité laissée. Le pourcentage par rapport à la prise habituelle est aisé à calculer puisque les enfants non malades ingèrent chaque jour un volume à peu près constant de nourriture connu des parents (même nombre de cuillères mesures de lait et même quantité d’eau selon les graduations du biberon pour un même repas).

La mesure de la SpO2 serait théoriquement un outil performant puisqu’elle est en relation directe par l’intermédiaire de la courbe de dissociation de l’hémoglobine, avec l’hypoxémie. L’hypoxémie est considérée comme le risque évolutif principal de la maladie. Mais cette mesure est délicate, nécessitant un temps prolongé chez un enfant calme, la certitude d’avoir un enregistrement suffisamment correct du pouls artériel. Le positionnement du capteur ne permet pas toujours d’obtenir une mesure, nécessitant parfois plusieurs tentatives. Les capteurs spécifiques aux nourrissons sont fragiles, vite hors d’usage, difficiles à désinfecter rigoureusement, coûteux pour un usage unique. Dans cette zone de mesure, une petite variation de la SpO2 est associée à une grande variation de la PaO2. Le résultat est aussi dépendant en partie de l’observateur et d’une certaine variabilité à 30 mn d’intervalle (14). Dans cette étude, tout résultat < 95% a été confirmé par une deuxième mesure en conservant la valeur la plus haute.

La valeur de 95% pour la SpO2 est un seuil habituellement utilisé pour définir une hypoxémie. Un taux prolongé de 90% est recommandé pour débuter une oxygénothérapie pour des enfants non à risque (prématuré, pathologies cardiaque ou pulmonaire et plus de 3 mois) (7).

Parmi les trois types de tirage, sus sternal, intercostal et sus costal, le tirage intercostal paraît être le signe le plus discriminant pour déceler une SpO2 < 95%.

Wang (13) constate que l’appréciation du tirage dépend en partie de l’examinateur, même professionnel, et de sa variabilité à 2 examens séparés de 10 à 30 mn. L’importance du tirage est difficilement quantifiable, ce qui pose le problème de l’interprétation des situations limites. Sa détection est malaisée pour des parents non initiés. Il est en général intégré dans des scores d’utilisation pas toujours facile à utiliser en pratique quotidienne. La mesure d’Alim 24h parait plus simple, plus facilement utilisable par les parents.

La décision d’hospitalisation était liée à l’âge < 2 mois (les recommandations en France préconisent d’hospitaliser tous les nourrissons de moins de 6 semaines) et à l’hypoxémie. L’hypoxémie étant liée à une Alim 24h < 50% et à la présence d’un tirage intercostal. En pratique, au cabinet, si la saturation ne peut être mesurée, en dehors de l’âge qui ne pose pas de difficulté d’interprétation, ces deux signes cliniques permettent d’orienter la décision d’hospitaliser.

Cette étude peut avoir plusieurs biais. C’est la même personne qui recueille toutes les données (Alim 24h, les signes cliniques de difficulté respiratoires, l’oxymétrie) et qui prends la décision d’hospitaliser. Le protocole a tenté de limiter la part subjective de ces appréciations en précisant l’ordre du recueil des données (les signes cliniques avant la mesure) et les précautions lors de la mesure de l’oxymétrie.

Ces résultats concernent des patients examinés en ville. D’autres études sur des enfants plus gravement malades (vus aux urgences, hospitalisés) seraient nécessaires pour valider ces résultats dans ces populations. Enfin, les enfants nourris au sein ont été exclus car la quantité de lait bue ne pouvait être estimée précisément, en particulier d’après la durée de la tétée.

Conclusion

Ce travail a étudié la valeur sémiologique d’un signe clinique pour évaluer la gravité d’une bronchiolite chez les nourrissons, non prématurés, bien portants auparavant, nourris essentiellement au biberon, examinés dans un cabinet médical de Pédiatrie.

Ce seul signe, Alim 24h, parait pertinent par la force de son caractère discriminant (hypoxémie ou non), par sa facilité d’évaluation, de mémorisation et d’utilisation possible par les parents. Il apparaît, en dehors du très jeune âge (moins de 6 semaines), comme une aide à un premier tri ainsi que pour suivre un enfant malade à distance. Si l‘alimentation des dernières 24 heures est au moins égale à la moitié de la quantité habituellement ingérée par l’enfant, celui-ci a peu de chance d’être hypoxémique. Si ce chiffre est inférieur à 50%, une consultation d’évaluation semble nécessaire.

Abréviations

ACTIV Association Clinique et Thérapeutique Infantile du Val de Marne, une association de recherche sur les pathologies infectieuses pédiatriques ambulatoires

Alim 24h Pourcentage de la prise alimentaire des dernières 24 heures par rapport à la quantité habituellement ingérée.

IC Intervalle de confiance

OD Odds ratio

RV+ Rapport de vraisemblance positif

RV – Rapport de vraisemblance négatif

SpO2 Saturation en oxygène par mesure transcutanée.

VPP Valeur prédictive positive

VPN Valeur prédictive négative

VRS Virus respiratoire syncitial

Déclaration de non conflit d’intérêt

Tous les auteurs déclarent n’avoir reçu aucune rémunération autre que celles de leur employeur. Ils déclarent ne pas avoir perçu d’intérêts non financiers, ne pas avoir eu de relation financière au cours des trois dernières années avec des sociétés commerciales qui auraient pu avoir un intérêt dans cette étude, tant pour eux-mêmes que pour leurs époux, partenaires ou enfants.

L’association ACTIV a été à l’initiative de cette étude. Les sources de financement d’ACTIV ne sont pas intervenues dans la conception de l’étude, la collecte des données, le travail d’analyse, la décision de publier et l’écriture de l’article.

Remerciements

Ce travail est dédié à la mémoire de Claudie Wollner.

Remerciements aux autres pédiatres investigateurs : Christian Copin, Patrice Deberdt, Solange Duriez, Geneviève Granat, Eric Sandrin, Anne-sylvestre Michot, Mohammed Benani, Allegra Brami, Jean Pierre Henon, Sydney Sebban, Marie Hélène A’kou, Eric Osika, à Michel Boucherat (ACTIV) qui a conçu la base de données, à Philippe d’Athis pour son assistance statistique, à Nicole Beydon pour sa relecture, à Manuela de Oliveira (ACTIV) pour son assistance technique.

Bibliographie

1- Carraro S, Zanconato S, Baraldi E. Bronchiolitis : from empirism to scientific evidence. Minerva Pediatr. 2009;61(2):217-25

2- [Perrotta C](http://www.ncbi.nlm.nih.gov/pubmed?term="Perrotta C"%5BAuthor%5D), [Ortiz Z](http://www.ncbi.nlm.nih.gov/pubmed?term="Ortiz Z"%5BAuthor%5D), [Roque M](http://www.ncbi.nlm.nih.gov/pubmed?term="Roque M"%5BAuthor%5D). Chest physiotherapy for acute bronchiolitis in paediatric patients between 0 and 24 months old. [Cochrane Database Syst Rev.](javascript:AL_get(this, 'jour', 'Cochrane Database Syst Rev.');) 2007 Jan 24;(1):CD004873.

3- [Gajdos V](http://www.ncbi.nlm.nih.gov/pubmed?term="Gajdos V"%5BAuthor%5D), [Katsahian S](http://www.ncbi.nlm.nih.gov/pubmed?term="Katsahian S"%5BAuthor%5D), [Beydon N](http://www.ncbi.nlm.nih.gov/pubmed?term="Beydon N"%5BAuthor%5D), [Abadie V](http://www.ncbi.nlm.nih.gov/pubmed?term="Abadie V"%5BAuthor%5D), [de Pontual L](http://www.ncbi.nlm.nih.gov/pubmed?term="de Pontual L"%5BAuthor%5D), [Larrar S](http://www.ncbi.nlm.nih.gov/pubmed?term="Larrar S"%5BAuthor%5D), [Epaud R](http://www.ncbi.nlm.nih.gov/pubmed?term="Epaud R"%5BAuthor%5D), [Chevallier B](http://www.ncbi.nlm.nih.gov/pubmed?term="Chevallier B"%5BAuthor%5D), [Bailleux S](http://www.ncbi.nlm.nih.gov/pubmed?term="Bailleux S"%5BAuthor%5D), [Mollet-Boudjemline A](http://www.ncbi.nlm.nih.gov/pubmed?term="Mollet-Boudjemline A"%5BAuthor%5D), [Bouyer J](http://www.ncbi.nlm.nih.gov/pubmed?term="Bouyer J"%5BAuthor%5D), [Chevret S](http://www.ncbi.nlm.nih.gov/pubmed?term="Chevret S"%5BAuthor%5D), [Labrune P](http://www.ncbi.nlm.nih.gov/pubmed?term="Labrune P"%5BAuthor%5D). Effectiveness of chest physiotherapy in infants hospitalized with acute bronchiolitis: a multicenter, randomized, controlled trial. [PLoS Med.](javascript:AL_get(this, 'jour', 'PLoS Med.');) 2010 Sep 28;7(9):e1000345.

4- Shaw KN, Bell LM, Sherman NH. Outpatient assessment of infants with bronchiolitis. AJDC 1991;145:151-5

5- Perlstein PH, Uma RK, Bolling C, Steele R, Schoettker PJ, Atherton HD, Farrell MK Evaluation of an evidence-based guideline for bronchiolitis. Pediatrics.1999;104(6):1334-41

6- Stagnara J, Balagny E, Cossalter B, Dommerges JP, Dournel C and al Prise en charge de la bronchiolite du nourrisson. Texte long des recommandations. Arch Pédiatr 2001;8 suppl. 1:11-23

7- American Academy of Pediatrics, Subcommittee on Diagnosis and Management of Bronchiolitis : Clinical practical guidelines. Diagnosis and management of bronchiolitis. Pediatrics 2006;118:1774-93

8- Dawson K, Kennedy D, Asher I, Cooper D, Cooper P, Francis P, et al. The management of acute bronchiolitis. J Paediatr Child Health 1993;29:335-7

9- Simoes EAF, King SJ, Lehr MV, Groothuis JR. Respiratory syncitial virus infection. Lancet 1999;354:847-52

10- Barben J, Hammer J. Traitement de la bronchiolite aigue du NRS. Pediatrica 2003:14(6)<http://www.swiss-paediatrics.org/paediatrica/vol14/n6/bronchiolite-fr.html>

11- Baumer JH. SIGN guideline on bronchiolitis in infants. Arch Dis Cild Pract Ed. 2007;92(5):ep149-51

12- Pinnington LL, Smith CM, Ellis RE, Morton RE. Feeding efficiency and respiratory integration in infants with acute viral bronchiolitis. J Pediatr 2000;137:523-6

13- Verges S, Bachasson D, Wuyam B. Effect of acute hypoxia on respiratory muscle fatigue in healthy humans. Respiratory research 2010;11:109

14- Wang EEL, Law BJ, Stephens D, Langley JM, MacDonald NE, Robinson JL, Dobson S, McDonald J, Boucher FD, de Carvalho V, Mitchel I. Study of interobserver reliability in clinical assessment of RSV lower respiratory illness. Pediatr Pulmonol. 1996;22:23-27

15- Riordan J, Gill-Hopple K, Angeron J: Indicators of effective breastfeeding and estimates of breast milk intake. *J Hum Lact* 2005, 21(4):406-412.


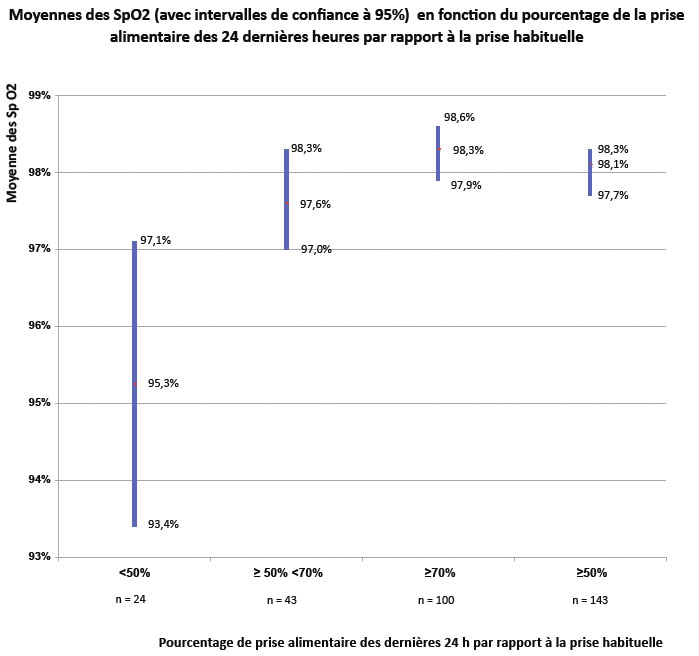


Figure1. Les trois rectangles de gauche montrent une variation dans le même sens de la moyenne des SpO2 et d’Alim 24h. Les premier et dernier rectangles témoignent de la différence significative des moyennes de SpO2 selon que l’enfant ingère < 50% ou ≥ 50% de sa ration habituelle au cours des dernières 24 heures. (4 données manquantes).

Figure 2. Alim 24h < 50% représente le meilleur compromis pour détecter une SpO2 < 95% avec une spécificité élevée (90 %) et une sensibilite moyenne (60 %).

**Tableau 1. Sensibilité, spécificité, VPP, VPN, RV^+^, RV^-^ et Odds Ratio des signes cliniques pour dépister une hypoxémie (SpO2 < 95%) au cours d'une bronchiolite.**

|  | Sensibilité  %  [IC 95%] | Spécificité  %  [IC 95%] | VPP  %  [IC 95%] | VPN  %  [IC 95%] | RV^+^  n  [IC 95%] | RV^-^  n  [IC 95%] | Odds ratio  n  [IC 95%] |
| --- | --- | --- | --- | --- | --- | --- | --- |
| Tirage sus sternal | 50  [21 - 79] | 87  [81 - 92] | 24  [9 - 45] | 96  [91 - 98] | 4  [2 - 8] | 0,6  [0,3 - 1] | 6.9  [2.0 - 23.7] |
| Tirage intercostal | 73  [45 - 92] | 80  [73 - 86] | 26  [14 - 42] | 97  [92 - 99] | 3,6  [2 - 6] | 0,3  [0,1 - 78] | 10.8  [3.2 - 36.3] |
| Tirage sous sternal | 47  [21 - 73] | 72  [64 - 79] | 14  [6 - 27] | 93  [87 - 97] | 1,7  [0,9 - 3] | 0,7  [0,5 - 1,2] | 2.3  [0.8 - 6.6] |
| Polypnée (≥ 50/min) | 87  [60 - 98] | 62  [53 - 70] | 19  [10 - 30] | 98  [93 - 100] | 2,3  [1,7 - 3] | 0,2  [0,06 - 0,8] | 10.5  [2.3 - 48.2] |
| Alim 24h < 70% | 87  [60 - 98] | 65  [56 - 72] | 19  [11 - 31] | 98  [93 - 100] | 2,4  [1,8 - 3,2] | 0,2  [0,06 - 0,8] | 11.8  [2.6 - 54.2] |
| Alim 24h < 60% | 67  [38-88] | 78  [70-84] | 23  [11 - 38] | 96  [91-99] | 3  [1,9 - 4,7] | 0,4  [0,2 - 0,8] | 6.9  [2.2 - 21.7] |
| Alim 24h < 50% | 60  [32 - 84] | 90  [84 - 94] | 38  [19 - 59] | 96  [91 - 99] | 6,1  [3 - 11] | 0,4  [0,2 - 0,8] | 13.8  [4.3 - 44.1] |
| Alim 24h < 40% | 33  [12-62] | 96  [92 - 99] | 46  [17 - 77] | 94  [89 - 97] | 8,5  [2,9 - 24] | 0,7  [0,5 - 0,99] | 12.2  [3.2 - 47.2] |

Les dénominateurs peuvent varier légèrement en fonction de quelques données manquantes.

Alim 24h : pourcentage de l’alimentation ingérée au cours des dernières 24 heures par rapport à d’habitude. VPP : Valeur prédictive positive, VPN : Valeur prédictive négative, RV+ : Rapport de vraisemblance positif, RV- : Rapport de vraisemblance négatif, [IC 95%] : Intervalle de confiance à 95%

| **Tableau 2.** Facteurs d'hospitalisation pour bronchiolite. | | | | |  |
| --- | --- | --- | --- | --- | --- |
|  |  |  |  |  |  |
|  | Hospitalisés (n=17) | | non hospitalisés (n=154) | |  |
|  | effectifs | % | effectifs | % | p |
| age < 2 mois | 13 | 76% | 7 | 5% | < 0,001 |
| Alim 24h < 50% | 9 | 53% | 15/150 (*) | 10% | < 0,001 |
| Tirage intercostal | 9 | 53% | 33/151 (*) | 22% | < 0,001 |
| 1 ou plus. de ces 3 facteurs | 14 | 82% | 49/147 (*) | 33% | < 0,001 |
|  |  |  |  |  |  |
| SpO2 < 95% | 11 | 65% | 4 | 3% | < 0,001 |
|  |  |  |  | * données manquantes | |
